# Increasing Prevalence of ESBL-Producing Multidrug Resistance *Escherichia coli* From Diseased Pets in Beijing, China From 2012 to 2017

**DOI:** 10.3389/fmicb.2019.02852

**Published:** 2019-12-10

**Authors:** Yanyun Chen, Zhihai Liu, Yaru Zhang, Zhenbiao Zhang, Lei Lei, Zhaofei Xia

**Affiliations:** ^1^Department of Veterinary Internal Medicine, College of Veterinary Medicine, China Agricultural University, Beijing, China; ^2^College of Chemistry and Pharmaceutical Sciences, Qingdao Agricultural University, Qingdao, China; ^3^Beijing Advanced Innovation Center for Food Nutrition and Human Health, College of Veterinary Medicine, China Agricultural University, Beijing, China; ^4^The New Hope Liuhe Co., Ltd., Qingdao, China

**Keywords:** multidrug resistance, antimicrobial drug usage, companion animals, ESBL, *Escherichia coli*

## Abstract

We investigated antimicrobial resistance trends and characteristics of ESBL-producing *Escherichia coli* isolates from pets and whether this correlates with antibiotic usage in the clinic. Clinical samples containing *E. coli* from diseased cats and dogs were screened for antibiotic sensitivity and associated genotypic features. We identified 127 *E. coli* isolates from 1886 samples from dogs (*n* = 1565) and cats (*n* = 321) with the majority from urinary tract infections (*n* = 108, 85%). High rates of resistance were observed for β-lactams and fluoroquinolones and resistance to > 3 antibiotic classes (MDR) increased from 67% in 2012 to 75% in 2017 (*P* < 0.0001). This was especially true for strains resistant to 6–9 antibiotics that increased from 26.67 to 60.71%. Increased rates in β-lactam use for clinical treatment accompanied these increasing resistance rates. Accordingly, the most frequently encountered subtypes were *bla*_CTX–M_ (*n* = 44, 34.65%), *bla*_CTX–M–65_ (*n* = 19) and *bla*_CTX–M–15_ (*n* = 18) and *qnrB* (*n* = 119, 93.70%). The *bla*_CTX–M_-isolates possessed 36 unique pulsed field electrophoretic types (PFGEs) and 28 different sequence types (STs) in ST405 (7, 15.9%), ST131 (3, 6.8%), ST73, ST101, ST372, and ST827 (2, 4.5% each) were the most prevalent. This data demonstrated a high level of diversity for the *bla*_CTX–M_-positive *E. coli* isolates. Additionally, *bla*_NDM–5_ was detected in three isolates (*n* = 3, 2.36%), comprised of two ST101 and one ST405 isolates, and *mcr-1* was also observed in three colistin-resistant *E. coli* with three different STs (ST6316, ST405, and ST46). Our study demonstrates an increasing trend in MDR and ESBL-producing *E. coli* and this correlated with β-lactam antibiotic usage for treatment of these animals. This data indicates that there is significant risk for the spread of resistant bacteria from pets to humans and antibiotic use for pets should be more strictly regulated.

## Introduction

Antimicrobial resistance has become one of the most challenging problems for public health and results in 700,000 deaths annually (O’Neill, 2016). Antibiotic misuse has led to the spread of antibiotic resistance genes (ARGs) in humans, food animals, pets, songbirds, water, and soil and even agricultural plants, and this represents a significant threat to public health security (Carter et al., 2018; Hartantyo et al., 2018; Anderson et al., 2019; Chen et al., 2019; Gros et al., 2019; Sanchez et al., 2019; Vikesland et al., 2019). However, novel ARGs have emerged that encode resistance to carbapenems (*bla*_NDM_, *bla*_IMP_, *bla*_VIM_, and *bla*_KPC_) (Perez and Bonomo, 2019) colistin (*mcr-1* to *9*) and tigecycline (*tet*X3 and *tet*X4) (Carroll et al., 2019; He et al., 2019; Wang et al., 2019). The current limited development of novel drugs and substitutes makes the use of ARGs monitoring even more important to develop comprehensive and integrative measures for antimicrobial resistance.

Over the past two decades, there has been a significant number of infections caused by bacteria expressing extended-spectrum-β-lactamase (ESBL) and carbapenemases (Logan and Weinstein, 2017). In particular, ESBL isolates have been found in humans (Pitout and Laupland, 2008), animals, the environment (water and soil) (Runcharoen et al., 2017), meat and even vegetables (Yang et al., 2019). ESBL are becoming more common because this phenotype is being selected for by the use and exposure to β-lactams, especially the cephalosporins. This has generated a vicious cycle of drug resistance and decreased therapeutic effects. The increasing use of cephalosporin has been linked to *Escherichia coli* infections in pigs (Hammerum et al., 2014) and a high frequency of ESBL-producing *E. coli* was directly linked with a high consumption of third- or fourth- cephalosporins (Andersen et al., 2015).

One area that has not been thoroughly investigated is ARG presence in companion animals. This group comes in intimate contact with humans and pet contact can lead to bacterial spread to humans (Lloyd, 2007). In particular, *E. coli* is a common pathogenic agent isolated from pets (Mathers et al., 2015) and is often present in dogs and cats with urinary tract infections (UTIs), pyometra and respiratory tract infections (Karkaba et al., 2019; Moyaert et al., 2019). Multidrug resistance (MDR) *E. coli* isolates have emerged in companion animals in the United States and Europe (Morrissey et al., 2016), but data for China is lacking.

Here, we explored the effects of antibiotic dose on resistance phenotypes in pet bacterial isolates over a 6-year period. The aim of this study was to investigate the dissemination of ESBL-producing multidrug-resistant pathogens in diseased pets and the correlation between resistance rates and consumption of β-lactam antibiotics.

## Materials and Methods

### Samples and Identification of Bacterial Isolates

Animal samples were collected at the Veterinary Teaching Hospital of China Agricultural University (VTH-CAU), between January 2012 and June 2017. This study was approved by the China Agricultural University Animal Ethics Committee and the approval document (No. AW08104102-2) (see Supplementary Information). We collected 1886 samples from dogs (1565) and cats (321) and where some samples were gathered from different infections in the same pet. All samples consisted of urine (UTI) samples (1398, 74.1%) and samples from pyoderma (125, 6.7%), ear swabs (6.1%), effusions (115, 6.1%) and other specimens (132, 7.0%), which included pus from the uterus and soft tissue infections and several trachea lavage fluids.

Bacterial strains were recovered using blood agar and MacConkey agar plates that were incubated at 37°C for 24 h and single pink colonies were collected from each isolation plate. Subsequently, the DNA of individual clones was extracted by Fast Pure Bacteria DNA Isolation Mini Kit (Vazyme Biotech, Nanjing, China) and used as templates for PCR. PCR amplification of the 16S rDNA gene were performed for all isolates as previously described (Brianna et al., 2013), and amplicons were sequenced to confirm bacterial genus using the BLAST algorithm^1^.

### Antimicrobial Susceptibility Testing

Antimicrobial susceptibility of isolates was performed using the broth microdilution method according to Clinical and Laboratory Standards Institute guidelines (CLSI, 2015a). The breakpoints for other antimicrobials used the CLSI (M100-S25 or Vet01-A4/Vet01-S2) and EUCAST (CLSI, 2015b; EUCAST, 2019), where the breakpoints (R) for tigecycline, orbifloxacin, enrofloxacin, and marbofloxacin were recommended as ≥0.5, ≥4, ≥4, and ≥8 μg/ml, respectively. The screening panel consisted of 17 antibiotics that included ampicillin, cefazolin, cefotaxime, ceftriaxone, meropenem, amoxicillin-clavulanic acid, aztreonam, ciprofloxacin, enrofloxacin, marbofloxacin, orbifloxacin, chloramphenicol, amikacin, gentamicin, doxycycline, colistin, and tigecycline. Isolates with resistance to three or more categories of antimicrobial agents were classified as MDR (Magiorakos et al., 2012). *E. coli* ATCC 25922 was used as the quality control strain. Resistance was categorized according to Standardized International Terminology and our 17 test antibiotics were contained within nine categories (Magiorakos et al., 2012).

### Survey of Antimicrobial Drug Usage at the VTH-CAU

Antibiotic usage at VTH-CAU was recorded between January 2014 and September 2017 to correlate antibiotic usage and the resistance of *E. coli* isolates for each study animal.

### ARG Detection

Screening of *E. coli* isolates for ARG types was conducted using PCR for each isolate depending on the antibiotic resistance phenotype. The ARGs we examined were (1) carbapenemase genes *bla*_NDM_, *bla*_IMP_ *bla*_KPC_, *bla*_VIM_, *bla*_OXA_, *bla*_AIM_, *bla*_BIC_, *bla*_DIM_, *bla*_GIM_, *bla*_SIM_, and *bla*_SPM_ including *bla*_NDM_ and *bla*_CTX–M_ subtyping (Poirel et al., 2011), (2) β-lactamase genes *bla*_SHV_, *bla*_TEM_, and *bla*_CTX–M_ (Casella et al., 2018), (3) plasmid-mediated AmpC β-lactamase genes *bla*_MOX_, *bla*_CMY_, *bla*_LAT_, *bla*_DHA_, *bla*_ACC_, *bla*_MIR_, *bla*_ACT_, and *bla*_FOX_ (Perez-Perez and Hanson, 2002), (4) colistin resistance genes *mcr-1*-*8* (Rebelo et al., 2018; Wang X. et al., 2018; Yang et al., 2018; Carroll et al., 2019), and (5) plasmid-mediated quinolone resistance (PMQR) genes *qnrA, qnrB* and *qnrS* including whether the *gyr*A and *par*C genes in the quinolone resistance determining region (QRDR) were mutated (Komp et al., 2003; Kraychete et al., 2016; Onseedaeng and Ratthawongjirakul, 2016). PCR primers used for screening are shown in Supplementary Table S1. All PCR amplicons were sequenced to confirm gene identity.

### Transconjugation Assays and Whole Genome Sequencing

Conjugation assays were performed between clinical isolates and *E. coli* J53 to evaluate whether *bla*_NDM_ and *mcr-1* were mobilizable. Transconjugants were selected on MacConkey agar containing 100 mg/L sodium azide and 1 mg/L meropenem or colistin. Presumptive transconjugants were identified using PCR screening for *bla*_NDM_ and *mcr-1*.

Transconjugant DNA was extracted and used for whole genome sequencing (WGS). A library of 250-bp paired-end was constructed by using a NEXT Ultra DNA Library Prep kit (New England Biolabs, Beverley, MA, United States) and sequenced using an Illumina HiSeq 2500 system at Bionova Biotech (Beijing, China). Raw data was *de novo* assembled using the SPAdes algorithm v.3.10.0. ARGs and plasmid incompatibility groups were analyzed using ResFinder v.3.2^2^, RASTtk v.2.0^3^ and PlasmidFinder 2.1^4^, respectively.

### PFGE and MLST Typing of *E. coli* Strains

The clonal relatedness of *bla*_CTX–M_ positive was determined using pulsed-field gel electrophoresis (PFGE) typing conducted as previously described (Tenover et al., 1995). PFGE patterns were visually inspected and gel images were analyzed using InfoQuest FP software (Biorad, Hercules, CA, United States). Group analysis of PFGE profiles was performed using the Dice coefficient and the unweighted pair group method with arithmetic means. Simultaneously, multilocus sequence typing (MLST) analysis was conducted using the following *E. coli* gene set: *recA, adk, fumC, icd, mdh, purA*, and *gyrB.* The results were interpreted using the MLST database^5^. Sequence types (STs) of 131 clades in *E. coli* isolates were also screened using multiplex conventional PCR assays as previously described (Matsumura et al., 2017).

### Statistical Analysis

Tests of statistical significance was determined using Fisher’s exact test with Yates continuity correction in GraphPad Prism 6 (San Diego, CA, United States) and the level of significance was set at *P* < 0.05. All figures were designed by ggplot2^6^ and GraphPad Prism 6.

## Results

### Samples and *E. coli* Isolates

We isolated 127 *E. coli* strains that included 108 (85.0%) urine and 5 (3.9%) uterus, 4 (3.1%) abdominal fluid, 3 (2.4%) pyoderma, 2 (1.6%) soft tissue infectious sites, and 1 (0.8%) from synovial fluid. The *E. coli* isolation rate was 6.73% (127/1886) and ranged from 4.42 to 9.41% per year. Resistance to β-lactams was extremely high for these isolates and included resistance to ampicillin (77.9%), cefotaxime (58.3%) and ceftriaxone (58.3%) and cefazolin (65.35%) with the exception of amoxicillin + clavulanic acid (22.8%) (Supplementary Tables S2, S3).

**FIGURE 1:**
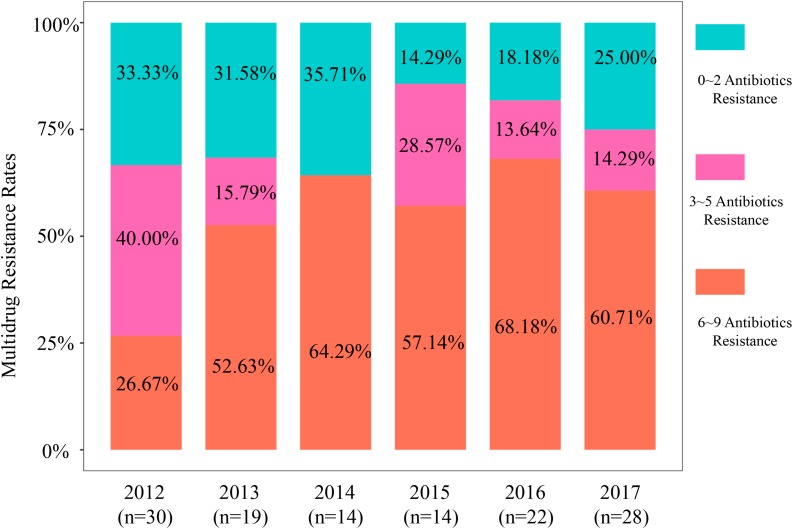
Multidrug resistance rates of 127 isolates based on antimicrobial category from 2012 to 2017.

The range of resistance rates was narrow for ampicillin (68.42–92.86%) compared with cefotaxime (36.67–72.73%), ceftriaxone and cefotaxime (36.67–72.73%), cefazolin (43.33–86.36%), and amoxicillin + clavulanic acid (10.00–27.27%). These data demonstrated a significant increase in resistance rates from 2012 to 2017 and the cefotaxime, ceftriaxone, cefazolin, and amoxicillin + clavulanic acid rates more than doubled (Supplementary Figure S1 and Supplementary Table S3). The aztreonam resistance rate showed the greatest variability and was maximal in 2014 (71.43%) and minimal in 2012 (13.33%). We also found high rates of resistance to the four fluoroquinolones we tested (52.76–57.48%) except for 2013 (36.84%) (Supplementary Table S3). The other resistance groups we examined displayed irregular trends or slight increases over the 2012–2017 study period. Notably, almost all isolates showed high susceptibility to the “last-resort” antibiotics colistin, meropenem, and tigecycline and only 5 (3.94%) of our isolates were resistant to colistin and 3 (2.4%) to meropenem (Figure 1 and Supplementary Table S3).

The MDR values for our isolates from these pets were high with an overall MDR rate that increased 73.2% from 2012 to 2017 (*n* = 93). These MDR rates from 2012 to 2017 were 66.67, 68.42, 64.29, 85.71, 81.82, and 75.00% in 2017, respectively (*P* < 0.0001) (Supplementary Table S4). Specifically, MDR prevalence in 6–9 antimicrobial categories exhibited an obvious increase (Figure 1 and Supplementary Table S4). The MDR of our 44 CTX-M-producing *E. coli* was greater than for the non-CTX-M-expressing isolates (Supplementary Figures S5a,b). Moreover, all *bla*_CTX–M_-positive *E. coli* were MDR strains possessing resistance to the penicillins [ampicillin (AMP)], non-extended spectrum cephalosporins [cefazoline (CZO)], extended-spectrum cephalosporins [cefotaxime (CTX) and ceftriaxone (CRO)]. The most common MDR pattern was resistance to quinolones (ciproxacin, enrofloxacin, orbifloxacin, and marbofloxacin) and the tetracyclines (doxycycline) and aminoglycosides (gentamycin) + β-lactams (AMP, CZO, CTX, and CRO). When this data was viewed solely by the number of antibiotics per MDR isolate, resistance rates to 11–17 antibiotics increased significantly over the study period from 13.3, 31.6, 35.7, 35.7, 50.0, and 53.6% from 2012 to 2017, respectively (Supplementary Figure S2 and Supplementary Table S5).

We performed a correlation analysis between antibiotic usage and the rate of antibiotic resistance between 2014 and 2017. Amoxicillin-clavulanic acid (1.3 × 10^7^ mg/yr), doxycycline (5.7 × 10^6^ mg/yr), ampicillin (1.9 × 10^6^ mg/yr), and enrofloxacin (8.1 × 10^5^ mg/yr) were the most widely used antibiotics at the animal facility. With the widespread use of FQs in VTH-CAU, >50% of the clinical *E. coli* isolates showed resistance to FQs since 2012. Interestingly, even though fluoroquinolone usage had decreased dramatically, resistance rates to this antibiotic class remained high. In addition, the use of β-lactams, including amoxicillin-clavulanic acid, ampicillin, and ceftriaxone, had increased over the study period and was positively correlated with an increase in β-lactam resistance that was also related to dosage. Meropenem and imipenem were not used at the facility and we found no resistance from 2012∼2015 although three resistant *E. coli* were detected in 2016 (Supplementary Figure S3).

### ARG Prevalence and NDM Plasmid Characterization

In our study, we identified 10 ARG types and mutations in *gyrA* and *parC*. The *qnrB* gene was present in 119 (93.70%) of the isolates (Supplementary Figure S4). In the *gyr*A gene of 77 quinolone-resistant isolates, 38 carried the mutations S83L and D87N, 9 S83L and D87Y, 2 D87N, 3 D87G, and 14 S83L (Supplementary Table S6). In the *par*C gene we found the mutations S80I (22 isolates), S80I and E84G (14 isolates) and S80I and E84V (8 strains). The ESBL and pAmpC-containing isolates harbored *bla*_CTX–M_ (*n* = 44, 34.65%), *bla*_SHV_ (*n* = 21, 16.55%), *bla*_OXA_ (*n* = 9, 7.09%), *bla*_CMY_ (*n* = 12, 9.45%), *bla*_FOX_ (*n* = 4, 3.15%), and *bla*_NDM_ (*n* = 3, 2.36%). CTX-M alleles were assigned to two main clusters; CTX-M-14 and -15 including seven CTX-M genotypes (-14, -15, -64, -65, -116, -127, and -174) where CTX-M-65 (43.18%, *n* = 19) and CTX-M-5 (40.91%, *n* = 18) predominated. All 44 *bla*_CTX–M_-positive isolates were resistant to cefotaxime and ceftriaxone and were classified as MDR. When compared with *bla*_CTX_-negative isolates, the *bla*_CTX_-positive isolates showed significantly greater resistance to second and third-generation cephalosporins, the fluoroquinolones, aztreonam, doxycycline, gentamicin, and chloramphenicol (*P* < 0.05) (Table 1 and Supplementary Figure S5). The only observed carbapenemase gene we identified was NDM-5 that was present in three isolates 16DU02, 16DF03, and 16XXI8 and all showed resistance to meropenem and all were collected from dogs in 2016. Of note, 16DU02 and 16DF03 were isolated from same dog but the samples were isolated from urine and an abdominal effusion, respectively, and they carried same genes *bla*_NDM–5_, *bla*_TEM_, *bla*_CTX–M–65_, and *qnrB*. The 16XXI8 isolate recovered from dog urine possessed *bla*_NDM–5_, *bla*_TEM_, *bla*_CTX–M–15_, *bla*_OXA_, and *qnrB*. Additionally, the colistin resistance gene *mcr-1* was detected in three *bla*_CTX–M_ positive *E. coli* while other *mcr* variants were undetected.

**TABLE 1:** Minimum inhibitory concentration (MIC) of antimicrobial agents for clinical *E. coli* isolates from cats and dogs in Beijing, China, 2012–2017 (*n* = 127)^‡^.

**Antimicrobial agents**	***bla***_**CTX**_***Positive E. coli* (*n* = 44)**			***bla***_**CTX**_**Negative *E. coli* (*n* = 83)**			***P*-value_†_**
	**MIC50 (μg/mL)**	**MIC90 (μg/mL)**	**Resistance, %**	**MIC50 (μg/mL)**	**MIC90 (μg/mL)**	**Resistance, %**	
Ampicillin	>128	>128	100.0	>128	>128	63.8	< 0.0001^∗∗^
Cefazolin	>128	>128	100.0	8	>128	47.0	< 0.0001^∗∗^
Cefotaxime	>128	>128	100.0	≤0.125	>128	31.3	< 0.0001^∗∗^
Ceftriaxone	>128	>128	100.0	≤0.125	>128	31.3	< 0.0001^∗∗^
Meropenem	≤0.125	≤0.125	6.8	≤0.125	≤0.125	0.0	0.0397^∗^
Amoxicillin-clavulanic acid	16/8	64/32	27.3	16/8	32/16	14.4	0.0972
Aztreonam	32	>128	75.0	≤0.125	>128	28.9	< 0.0001^∗∗^
Colistin	≤0.125	≤0.125	6.8	≤0.125	≤0.125	1.2	0.1195
Doxycycline	16	32	59.1	8	32	32.5	0.0048^∗∗^
Tigecycline	≤0.125	≤0.125	0.0	≤0.125	≤0.125	0.0	1.0000
Gentamycin	>128	>128	77.3	8	>128	36.1	< 0.0001^∗∗^
Amikacin	8	>128	27.3	8	>128	13.2	0.0575
Chloramphenicol	16	>128	50.0	8	128	24.1	0.0052^∗^
Ciprofloxacin	16	>128	81.8	0.25	64	37.3	< 0.0001^∗∗^
Enrofloxacin	32	128	81.8	0.25	64	39.7	< 0.0001^∗∗^
Orbifloxacin	128	>128	81.8	4	>128	38.6	< 0.0001^∗∗^
Marbofloxacin	16	64	81.8	0.5	32	37.3	< 0.0001^∗∗^

*^‡^The 127 isolates consisted of 107 isolates from urinary tract, 5 from uterus, 4 from abdominal fluid, 1 from synovial fluid, and 10 from pyoderma and soft tissue. ^†^*P-*values were determined by Fisher’s exact test. ^∗^*P* < 0.05; ^∗∗^*P* < 0.01.*

We further examined our *mcr-1* and *bla*_NDM_ isolates and tested whether these genes were present on mobile elements. In our conjugation tests, only *bla*_NDM_ in three *E. coli* isolates were successfully transferred at frequencies of 4.86 × 10^–8^–8.02 × 10^–7^. Two complete 46,161 bp *bla*_NDM–5_-harboring plasmids pP16NDM-502 (MN701974) and pP16NDM-503 (MN701975) were obtained from strains 16DU02 and 16DF03 transconjugants, respectively. The backbone sequences were assembled and contigs and gaps were identified by additional PCR and sequence analyses. The other NDM-1-carrying plasmid was unsuccessfully assembled because of fragmentary and short contigs (Supplementary Figure S6). The two completely assembled plasmids were all in the IncX3 replication group. In addition, *bla*_NDM–5_ was contained within an insertion sequence (IS) cassette (ΔISA*ba125*-IS*5*-*bla*_NDM_-*ble*-*trpF*-*dsbC*-IS*26*). *bla*_NDM–5_ and *ble*_MBL_ were the only ARGs present in the two plasmids. Homology analysis revealed that pP16NDM-502 and pP16NDM-503 were ≥ 99% identical to the following IncX3 *bla*_NDM_ plasmids: (i) pNDM_MGR194 (KF220657) from a *Klebisella pneumoniae* human isolate in India, (ii) p1079-NDM (MG825384) from a chicken *E. coli* isolate in China. (iii) pL65-9 (CP034744) from *E. coli* in goose in China, (iv) pZHDC40 (KY041843) from *E. coli* human isolate in China, (v) pQDE2-NDM (MH917280) from *K. pneumoniae* human isolate in China, (vi) pCRCB-101_1 (CP024820) from a *Citrobacter freundii* human isolate in Korea (vii) p128379-NDM (MF344560) from *Enterobacter hormaechei* from a human sample in China and (viii) pAD-19R (KX833071) from a chicken *E. coli* isolate in China (Supplementary Figure S6). We were unable to transfer the *mcr*-1 gene by conjugation in three separate tests and we could not assemble a complete plasmid sequence. However, no complete plasmid sequence of *mcr-1* was successfully assembled by using three clinical isolates genomes. Although the analysis of incompatibility group in three clinical isolates genomes by analyzed PlasmidFinder-2.0 Serve revealed they possessed IncFIB, IncFIC, IncFII, and IncHI2 typical fragments, all *mcr-1* genes weren’t located on those fragments (data not showed).

### PFGE and MLST Typing

**FIGURE 2:**
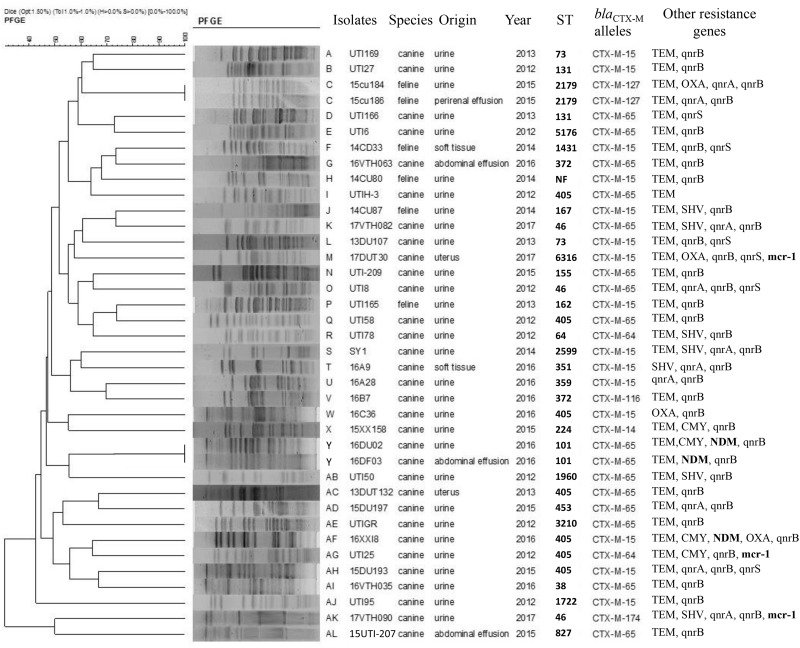
*Xba*I pulsed-field gel electrophoresis (PFGE) analysis of *E. coli* containing *bla*_CTX_ variants.

In our group of 127 *E. coli* isolates, most (*n* = 109, 85.8%) were successfully characterized by PFGE typing and included 38/44 *bla*_CTX–M_-positive isolates. These 38 isolates were obtained from cats (6) and dogs (32) and could be subdivided into 36 unique PFGE patterns. Interestingly, two isolates obtained from urine (15cu184) and perirenal effusion (15cu186) samples showed identical PFGE patterns and carried the same *bla*_CTX–M–127_ subtype but were recovered from different animals (Figure 2).

**FIGURE 3:**
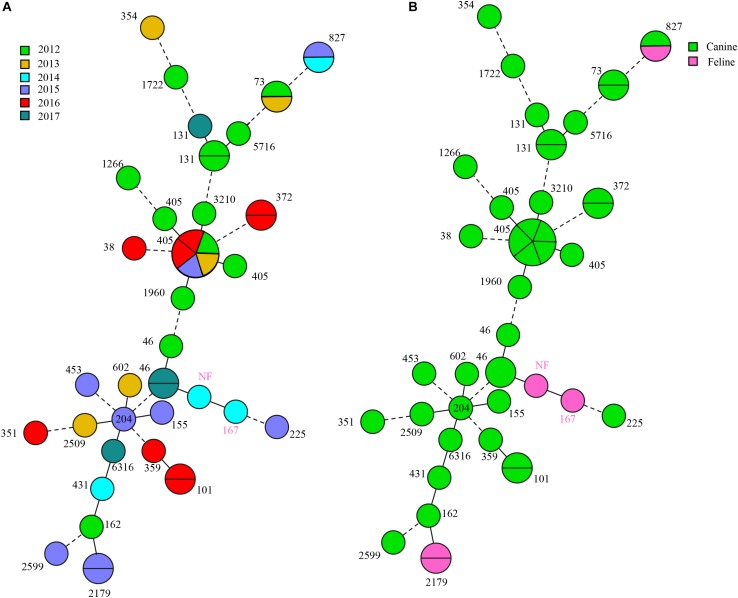
*bla*_CTX–M_-positive *E. coli* MLST typing. **(A)** MLST type colored based on different years. **(B)** MLST type colored based on different origins.

We also found universality in MLST types and identified 28 different STs although 1 isolate failed to type. The most prevalent were ST405 (7, 15.9%), ST131 (3, 6.8%) ST73, ST101, ST372, and ST827 (2, 4.5% each). All ST405 strains were collected from dogs but in different years. Three ST131 isolates (UTI-27, UTI-166, and DU40) belonged to clade C1, in which UTI-27 and DU40 were assigned as C1-nM27, and UTI-166 as C1-M27. The *bla*_CTX–M–65_ and *bla*_CTX–M–15_ positive *E. coli* exhibited the greatest ST diversity and contained 13 and 14 STs, respectively. Additionally, three *bla*_NDM–5_-positive isolates belonged to ST405 (*n* = 1) and ST101 (*n* = 2) and three carrying-*mcr-1 E. coli* were classified as three different STs: ST6316, ST405, and ST46 (Figure 3).

## Discussion

In this study, we investigated the antibiotic resistance profiles and trends in MDR *E. coli* isolates collected from diseased dogs and cats. We identified 127 *E. coli* isolates and most were associated with UTI (84.2%) accounting for 20.4% (108/529) of the confirmed bacterial UTI cases. In contrast, in the United States nearly 30% of UTI isolates from pets were *E. coli* (Ling et al., 2001; Hall et al., 2013). Previous studies in European countries based on 22,256 isolates from dogs and cats with UTI between 2008 and 2013 showed that *E. coli* was the most common pathogen in both dogs (59.50%) and cats (59.31%) (Marques et al., 2016). Isolation of *E. coli* from the respiratory tract is much less frequent and occurred in 10–15% of dog respiratory tract infections (Rheinwald et al., 2015; Morrissey et al., 2016).

Our 127 clinical *E. coli* isolates showed high prevalence rates of resistance to β-lactams (58.3–77.9%) and quinolones (52.8–57.4%). This pattern was quite different from the data of dog and cat *E. coli* isolates from United States that identified resistance to ampicillin at 40%, cephalexin 98% and doxycycline at 100% (Thungrat et al., 2015). An Australian study revealed that canine clinical *E. coli* isolates had low rates of resistance to quinolones (9.1–9.3%) and among 392 canine UTI isolates, 9.9–10.2% were resistant to third-generation cephalosporins (Saputra et al., 2017).

In the current study, the overall MDR frequency of 73.2% was higher than that observed in studies from the United States (52%) and Poland (66.8%) (Rzewuska et al., 2015; Thungrat et al., 2015). In contrast, a European multi-centre study on AMR of various bacteria isolated from companion animals with UTIs showed a much lower frequency of MDR among *E. coli* isolates (1.4–29.7%). The high MDR rates observed in the current study indicated that currently available antimicrobial treatment options for *E. coli* infections in companion animals are limited. We also identified an increase in *E. coli* MDR prevalence between 2012 and 2017 and increased rates of third-generation cephalosporins and amoxicillin-clavulanic acid usage was accompanied by increased AMR and MDR rates. Based on these findings, we speculate that the rising incidence of multidrug-resistant *E. coli* might be associated with the heavy use of these antibiotics in companion animals at the hospital.

Among the 44 CTX-M-producing *E. coli* isolates in this study, *bla*_CTX–M–65_ (43.2%) and *bla*_CTX–M–15_ (40.9%) were the most *bla*_CTX–M_ variants. Currently, more than 220 CTX-M-lactamases have been reported and clustered into five subgroups, containing CTX-M-1, -2, -8, -9, and -25 depending on amino acid sequence homology (Peirano and Pitout, 2019). CTX-M-15 (CTX-M-1) is the most frequent CTX-M variant worldwide (Karim et al., 2001) especially in South-East Asia, China, South Korea, and Japan (Peirano and Pitout, 2019). Since 2000, *bla*_CTX–M–15_ has emerged worldwide and is the most prevalent ESBL globally (Peirano and Pitout, 2019). The spread of *bla*_CTX–M–15_ contributed to the dissemination of MDR bacterial isolates in animals but also in humans; a significant public health concern (Mugnaioli et al., 2006; Canton et al., 2012; Liu et al., 2016b). While the *bla*_CTX–M–15_ variant was also common in canine *E. coli* isolates from both Shanxi, China (*n* = 40) and the United States (*n* = 50), *bla*_CTX–M–123_ predominated in those studies (Liu et al., 2016a, b). The *bla*_CTX–M–65_ was the most prevalent subtype we found and is frequently detected in *Salmonella* isolates. In a previous study, the *bla*_CTX–M–65_ gene (*n* = 131) was identified among 153 ESBL-positive *Salmonella* isolates from poultry slaughterhouses (*n* = 121) and humans (*n* = 10) (Bai et al., 2016). Similarly, a study on chickens and pigs in China surveyed seven *bla*_CTX–M–65_ among *Salmonella* isolates (Zhang et al., 2016). All these studies indicated more frequent occurrences in China and is supported by data from 2005 to 2017 in another study (Bevan et al., 2017). Additionally, *bla*_CTX–M–55_ prevalence has increased in recent years in China among animal and human isolates, although not in our present study. Conversely, *bla*_CTX–M–14_ has a remarkable reduction based on data for China (Bevan et al., 2017). We found only one *bla*_CTX–M–14_ isolate while this gene is globally present (Bevan et al., 2017). Although other *bla*_CTX–M_ variants, such as *bla*_CTX-M-64_, _-174_, _-116_, and _-127_ rarely occur, their presence in diseased pets suggests that diversity and evolution of *bla*_CTX–M_ had occurred in these companion animals.

All *bla*_CTX_-carrying isolates in the current study had MDR profiles and showed high resistance rates to β-lactams, quinolones, doxycycline, gentamycin, and chloramphenicol. This suggested that other resistance genes may be co-transferred with *bla*_CTX_, making it even more difficult to eliminate the spread of MDR. Most *bla*_CTX–M_ positive strains in other studies were also resistant to quinolones due to topoisomerase modifications of *qnr* genes (Lahlaoui et al., 2014) as we found in the present study. Additionally, mutations in *gyrA* and *parC* also can be responsible for quinolone resistance. Our results showed S83L and D87 alterations to N, Y, G generated in *gyrA* were commonly associated with resistance to fluoroquinolones and mostly generate high level resistance (Basu and Mukherjee, 2019).

Only three *E. coli* isolates containing *bla*_NDM–5_ were identified in our study animals with UTI in late 2016. Five (3.94%) were colistin resistance and three carried *mcr-1*. Previous reports implied that the transmissible *bla*_NDM_ and *mcr-1*-carrying plasmids play a major role in the dissemination of these genes (Wang et al., 2017). For example, *mcr-1*, initially named as mobile colistin resistance gene, was generally considered as mediating the rapid spread of bacterial colistin resistance worldwide due to its mobile plasmid association. In contrast, we found that *mcr-1* could not be mobilized by conjugation. In a previous study, 14/23 *mcr-1*-positive isolates were successfully transferred and six plasmids were non-transferable (Zhou et al., 2017). The *mcr-1* gene was located in the chromosome of *E. coli* from a goose isolate (Lu et al., 2019). Furthermore, IS*Apl1* transposon can mediate *mcr-1* transfer from chromosome to plasmids and this is the reason for its current global distribution (Wang R. et al., 2018). So, *mcr-1*-carried in a non-transferable plasmid or chromosome may be spread by IS*Apl1* or others transposons. In our study, we could not assemble a complete plasmid sequence for the *mcr-1* genomic data; and *mcr-1* genes were not present on those plasmid fragments and may be chromosomal. There are nine variants of *mcr* and we identified only *mcr-1* (Carroll et al., 2019). This gene is the most prevalent variant globally and has been detected in almost 40 countries/regions across five continents in both involving developed and non-developed countries. Moreover, the *mcr-1* gene was found in more than 11 bacterial species and in diverse locations such as rivers, public beaches, well water, wastewater, hospital sewage, foods (vegetables and meats), animals (wild birds, housefly/blowfly, cattle, pigs, poultry, and companion animals) (Feng, 2018). Our study provided new evidence for the above claim and we identified three *mcr-1* positive *E. coli* in diseased pets. Recently, carbapenem-resistant Enterobacteriaceae (CRE) have posed a threat to humans and animals because they exhibited resistance to most β-lactams including carbapenems, further compromising treatment of MDR infections (Gupta et al., 2011; Potter et al., 2016).

Carbapenem-resistant Enterobacteriaceae is mediated largely by the production of carbapenemase especially for NDM isolates. NDM-5, an NDM-1 variant, exhibited increased enzyme activity to carbapenems (Rogers et al., 2013) and its gene *bla*_NDM–5_ has been reported worldwide (Khan et al., 2017) and is the most prevalent variant in China (Shen et al., 2018). In support of this, 84 (52%) NDM-5-producing *E. coli* were collected from 161 *bla*_NDM_ carrying CRE in chickens (Wang et al., 2017). Our study revealed that pets have become a reservoir of NDM-5-producing *E. coli*. The *bla*_NDM–5_ gene is also associated with different plasmids such as IncFIA/B, IncFII, IncN, and IncX3 (Sun et al., 2015; Tyson et al., 2019; Zhang et al., 2019) and IncX3 was the dominant type (Li et al., 2018; Zhang et al., 2019). Similarly, the *bla*_NDM–5_-harboring plasmid was assigned to the IncX3 type, and their transferability was confirmed in our study indicating a risk of *bla*_NDM–5_ plasmid transfer between bacteria. However, further studies should be conducted to determine the origins of *bla*_NDM_ and *mcr-1* in the *E. coli* isolates from companion animals at the hospital. This will be helpful in designing measures to control the spread of MDR isolates.

All CTX-M-producing *E. coli* in our study displayed a diversity of PFGE patterns and STs demonstrating that *bla*_CTX–M_ encoding ESBLs are present in diverse *E. coli*. Additionally, our diseased pet samples were from animals having no prior contact suggesting that clonal spread had a low frequency and it was not likely that clonal spread of *bla*_CTX–M_-positive *E. coli* occurred between the pets. But ESBL or other resistance genes can transfer between different *E. coli* by transferable genetic elements, as previously reported (Kim et al., 2019). However, two *E. coli* isolates (15cu184 and 15cu186) from same cat but different sample sources shared the same PFGE, ST, and *bla*_CTX–M–127_ types, indicating that clonal spread may be occurring. It was reported that dissemination of Enterobacteriaceae can occur between pets and their owners by both horizontal transfer and clonal expansion (Yao et al., 2016).

In our study, ST405 *E. coli* (*n* = 7) was the most prevalent isolates carrying *bla*_CTX–M_. They presented different *bla*_CTX–M_ subtype genes including *bla*_CTX–M–15_ (*n* = 3), *bla*_CTX–M–65_ (*n* = 3), and *bla*_CTX–M–64_ (*n* = 1). In a clinical study from King Abdulaziz Medical City (KAMC) in Riyadh (Alghoribi et al., 2015), six ST405 *E. coli* harboring *bla*_CTX–M_ were detected in UTI and ST405 (*P* ≤ 0.02) were significantly associated with ESBL production. ST405 extended-spectrum β-lactamase-producing *E. coli* (ESBL-EC) was also detected in animals, in which one ST405 ESBL-EC was screened in barbary macaques (*Macaca sylvanus*) in Algeria, notably, it held the *mcr-1*, *bla*_TEM–1_, and *qnrB19* genes and *bla*_CTX–M–15_ (Bachiri et al., 2017). We observed a similar pattern and a UTI25 (ST405 ESBL-EC) isolate harbored *mcr-1*, *bla*_TEM_, *qnrB*, and *bla*_CTX–M–64_. Additionally, ST405 ESBL-EC (16XXI8) carried *bla*_NDM–5_, *bla*_OXA_, *bla*_TEM_, and *qnrB*. These data indicated ST405 ESBL-EC has become the vector of multiple resistance genes, containing carbapenem- and colistin-resistance genes. In a Sweden project, ST405 ESBL-EC was also found in UTI from a diseased cat (Bogaerts et al., 2015). We found seven ST405 ESBL-EC that were all recovered from dogs but in different years. Thus, companion animals already contain the hosts of ST405 ESBL-EC. In addition, except for ST405, we also identified two ST101 *E. coli* carrying *bla*_NDM–5_ (16DU02 and 16DF03). They were from the same animal source and possessed the same ST, PFGE, plasmid type and *bla*_NDM–5_, suggesting a clonal origin. ST101 was reported to be strongly associated with the NDM genotype although most were NDM-1 rather than NDM-5. Recently, five *bla*_NDM–5_-positive *E. coli* were identified associated with ST101 and ST1196 (Ranjan et al., 2016; Aung et al., 2018). In contrast, only two ST101 NDM-5-producing *E. coli* were observed in another study and most were ST167 and ST410 but were clonally spread in a hospital (Sun et al., 2019). This was similar to our study and suggested that ST101 may be becoming the most important clone for dissemination of *bla*_NDM–5_, similar to ST167 and ST410.

The clone ST131 was observed in three *E. coli* isolates. This is the predominant *E. coli* lineage in extraintestinal pathogenic *E. coli* (ExPEC) isolates worldwide and are associated with global community and nosocomial dissemination (Marie-Hélène et al., 2014). Currently, ST131 is classified as three clades: A, B, and C (Peirano and Pitout, 2019). In our study, all ST131 belonged to clade C and ST131 is in clade C 80% of the time (Peirano and Pitout, 2019). Clade C evolved from clade B and further evolved into C1 and C2. Recently, a new C1 subclade C1-M27 was identified in animal and human (Matsumura et al., 2016), including companion animals (Melo et al., 2019), and we found one C1-M27 subclades that are rare in China, but prevalent in Europe (Ghosh et al., 2017; Merino et al., 2018). The other ST131 belonged to subclade C1-nM27. C1 is commonly associated with quinolone resistance, in agreement with our results. The ESBL-EC isolates were commonly associated with ST131, where 40–80% of ESBL ExPEC belong to ST131 *E. coli* (Marie-Hélène et al., 2014), and CTX-15 was the most prevalent ESBL enzyme in ST131 ESBL-EC (Alghoribi et al., 2015). Our study demonstrated that *bla*_CTX–M–65_ was found in two ST131 ESBL-EC and one *bla*_CTX–M–15_ isolate. Almost all ST131 isolates were resistant to fluoroquinolones, especially for ESBL CTX-M-15 isolates (Marie-Hélène et al., 2014). We found similar results that almost all CTX-M ESBL isolates (40/44) were resistant to quinolones. ST131 ESBL *E. coli* possessed more frequent resistance to amikacin than non-ST131 ESBL isolates, but showed more frequent susceptibility to gentamicin or trimoxazole. On the other hand, the non-ESBL isolates such as *E. coli* ST131 were more frequently resistant to fluoroquinolones than non-ST131 isolates. Therefore, the resistance to quinolones may be associated with ST131 rather than ESBL presence (Marie-Hélène et al., 2014). These findings indicated that quinolones resistance may have been the predecessor of ESBL enzymes. We found that ST131 and ST405 were correlated to ESBL production as previously found (Alghoribi et al., 2015). Other STs in our study occurred infrequently. Recently, a water sample study revealed that ESBL-producing *E. coli* isolates were present 15 different STs (ST10, ST46, ST48, ST58, ST69, ST101, ST117, ST131, ST141, ST288, ST359, ST399, ST405, ST617, and ST4530) (Said et al., 2016), We detected ST46, ST101, ST131, ST359, and ST405 in our study. Multiple ST types (ST46, ST1286, ST10, ST29, ST101, and ST354) have also been found in chickens and they carried *mcr-1* and produced ESBLs (Wu et al., 2018). In our work, 28 diverse STs in ESBL-EC were found.

## Conclusion

We found a high prevalence of MDR *E. coli* isolates from diseased cats and dogs in Beijing and this rate has markedly increased over the last 6 years. The widespread use of third-generation cephalosporins and amoxicillin-clavulanic acid at the veterinary teaching hospital has likely contributed to the increasing frequency of β-lactam resistance in these isolates. These strains carried *bla*_CTX_, *bla*_NDM–5_, and *mcr-1* and most possessed an MDR profile. Diversity analysis of the PFGE patterns and STs of these clinical *E. coli* isolates from different origins suggested that the dissemination of *bla*_CTX_ have broad reservoirs of *E. coli*. Future studies should be undertaken to identify the MDR transmission mechanisms and establish national standards for the rational use of antibiotics in companion animals.

## Data Availability Statement

All datasets generated for this study are included in the article/Supplementary Material.

## Ethics Statement

This study was approved by the Agricultural University Animal Ethics Committee, China. The animals were given the best practice veterinary care and informed consent was granted by the owners.

## Author Contributions

ZX was responsible for the study design. YC, ZL, YZ, and ZZ assisted in the data collection. ZL, YC, and LL interpreted the data. ZL, YC, and ZX completed the report writing. All authors revised, reviewed, and approved the final report.

## Conflict of Interest

YZ was employed by the company of Shandong New Hope Liuhe Group Ltd. The remaining authors declare that the research was conducted in the absence of any commercial or financial relationships that could be construed as a potential conflict of interest.

## Footnotes

1 https://www.ncbi.nlm.nih.gov/

2 https://cge.cbs.dtu.dk

3 http://rast.nmpdr.org/rast.cgi

4 https://cge.cbs.dtu.dk/services/PlasmidFinder/

5 http://enterobase.warwick.ac.uk/species/ecoli/allele_st_search

6 https://ggplot2.tidyverse.org/
