# Supplementary material for: Increasing Prevalence of ESBL-Producing Multidrug Resistance Escherichia coli From Diseased Pets in Beijing, China From 2012 to 2017
Source: Front Microbiol. 2019 Dec 10;10:2852. doi: 10.3389/fmicb.2019.02852 (PMC6915038; doi:10.3389/fmicb.2019.02852)

**Table S1.** Primers used in this study

| **Primer name** | **Sequence (5'--3')** | **genes** | **Reference** |
| --- | --- | --- | --- |
| CTX-M-G1-F | TTAGGAARTGTGCCGCTGYA | *bla*CTX-M group 1 | (Casella et al., 2018) |
| CTX-M-G2-F | CGTTAACGGCACGATGAC | *bla*CTX-M group 2 |  |
| CTX-M-G1/2-R | CGATATCGTTGGTGGTRCCAT | *bla*CTX-M groups 1 and 2 |  |
| CTX-M-G9-F | TCAAGCCTGCCGATCTGGT | *bla*CTX-M group 9 |  |
| CTX-M-G9-R | TGATTCTCGCCGCTGAAG |  |  |
| CTX-M8/25-F | AACRCRCAGACGCTCTAC | *bla*CTX-M group 8/25 |  |
| CTX-M8/25-R | TCGAGCCGGAASGTGTYAT |  |  |
| SHV-F | AGCCGCTTGAGCAAATTAAAC | *bla*SHV |  |
| SHV-R | ATCCCGCAGATAAATCACCAC |  |  |
| TEM-F | ATGAGTATTCAACATTTCCG | *bla*TEM |  |
| TEM-R | CCAATGCTTAATCAGTGAGG |  |  |
| CTX-M15/55-F | AAAATGATTGAAAGGTGGT | sequencing of *bla*CTX-M-15 and -55 |  |
| CTX-M15/55-R | CAGCGCTTTTGCCGTCTAAG |  |  |
| CTX-M-2-F | ATGATGACTCAGAGCATTCG | sequencing of *bla*CTX-M-2 |  |
| CTX-M-2-R | TCAGAAACCGTGGGTTAC |  |  |
| CTX-M-8-F | CGGATGATGCTAATGACAAC | sequencing of *bla*CTX-M-8 |  |
| CTX-M-8-R | GTCAGATTGCGAAGCGTC |  |  |
| CTX-M-14-f | ATGGTGACAAAGAGAGTGCAA | *bla*CTX-M-14 | This study |
| CTX-M-14-r | TTACAGCCCTTCGGCGATGAT |  |  |
| CTX-M-174-f | ATGGTGACAAAGAGAGTGCTA | *bla*CTX-M-174 |  |
| CTX-M-174-r | TTACAGCCCTTCGGCGATGAT |  |  |
| CTX-M-64-f | ATGGTTAAAAAATCACTGCG | *bla*CTX-M-64 |  |
| CTX-M-64-r | TTACAAACCGTCGGTGACGA |  |  |
| CTX-M-116/127-f | ATGGTTAAAAAATCACTGCG | *bla*CTX-M-116 |  |
| CTX-M-116/127-r | TTACAAACCGTTGGTGACGA |  |  |
| mcr-9-f | ATGCCTGTACTTTTCAGGGTG | *mcr-9* | This study |
| mcr-9-r | TTAGCCACGGCATTCGCGGA |  |  |
| mcr-8-f | ATGTTCAAGTATCTTTTAT | *mcr-8* |  |
| mcr-8-r | TAACCATTCCCATCTGTTT |  |  |
| mcr-7-f | GTGCTCTGGTCGCTGAAGGT | *mcr-7 1119* |  |
| mcr-7-r | GCGATTGTCTGCCGCTTT |  |  |
| mcr-6-f | ATGACACAGCATAGTCCTTGG | *mcr-6* |  |
| mcr-6-r | TCAGCGGATGAATGCGGTGCG |  |  |
| mcr1_320bp_fw | AGTCCGTTTGTTCTTGTGGC | *mcr-1* | (Rebelo et al., 2018) |
| mcr1_320bp_rev | AGATCCTTGGTCTCGGCTTG |  |  |
| mcr2_700bp_fw | CAAGTGTGTTGGTCGCAGTT | *mcr-2* |  |
| mcr2_700bp_rev | TCTAGCCCGACAAGCATACC |  |  |
| mcr3_900bp_fw | AAATAAAAATTGTTCCGCTTATG | *mcr-3* |  |
| mcr3_900bp_rev | AATGGAGATCCCCGTTTTT |  |  |
| mcr4_1100bp_fw | TCACTTTCATCACTGCGTTG | *mcr-4* |  |
| mcr4_1100bp_rev | TTGGTCCATGACTACCAATG |  |  |
| MCR5_fw | ATGCGGTTGTCTGCATTTATC | *mcr-5* |  |
| MCR5_rev | TCATTGTGGTTGTCCTTTTCTG |  |  |
| IMP-F | GGAATAGAGTGGCTTAAYTCTC | *bla*IMP | (Poirel et al., 2011) |
| IMP-R | GGTTTAAYAAAACAACCACC |  |  |
| SPM-F | AAAATCTGGGTACGCAAACG | *bla*SPM |  |
| SPM-R | ACATTATCCGCTGGAACAGG |  |  |
| AIM-F | CTGAAGGTGTACGGAAACAC | *bla*AIM |  |
| AIM-R | GTTCGGCCACCTCGAATTG |  |  |
| VIM-F | GATGGTGTTTGGTCGCATA | *bla*VIM |  |
| VIM-R | CGAATGCGCAGCACCAG |  |  |
| OXA-F | GCGTGGTTAAGGATGAACAC | *bla*OXA-48 |  |
| OXA-R | CATCAAGTTCAACCCAACCG |  |  |
| GIM-F | TCGACACACCTTGGTCTGAA | *bla*GIM |  |
| GIM-R | AACTTCCAACTTTGCCATGC |  |  |
| BIC-F | TATGCAGCTCCTTTAAGGGC | *bla*BIC |  |
| BIC-R | TCATTGGCGGTGCCGTACAC |  |  |
| SIM-F | TACAAGGGATTCGGCATCG | *bla*SIM |  |
| SIM-R | TAATGGCCTGTTCCCATGTG |  |  |
| NDM-F | GGTTTGGCGATCTGGTTTTC | *bla*NDM |  |
| NDM-R | CGGAATGGCTCATCACGATC |  |  |
| DIM-F | GCTTGTCTTCGCTTGCTAACG | *bla*DIM |  |
| DIM-R | CGTTCGGCTGGATTGATTTG |  |  |
| KPC-Fm | CGTCTAGTTCTGCTGTCTTG | *bla*KPC |  |
| KPC-Rm | CTTGTCATCCTTGTTAGGCG |  |  |
| MOXMF | GCTGCTCAAGGAGCACAGGAT | *bla*MOX-1, *bla*MOX-2, *bla*CMY-1, *bla*CMY-8 to CMY-11 | (Perez-Perez et al., 2002) |
| MOXMR | CACATTGACATAGGTGTGGTGC |  |  |
| CITMF | TGGCCAGAACTGACAGGCAAA | *bla*LAT-1 to *bla*LAT-4, *bla*CMY-2 to *bla*CMY-7 |  |
| CITMR | TTTCTCCTGAACGTGGCTGGC |  |  |
| DHAMF | AACTTTCACAGGTGTGCTGGGT | *bla*DHA-1, *bla*DHA-2 |  |
| DHAMR | CCGTACGCATACTGGCTTTGC |  |  |
| ACCMF | AACAGCCTCAGCAGCCGGTTA | *bla*ACC |  |
| ACCMR | TTCGCCGCAATCATCCCTAGC |  |  |
| EBCMF | TCGGTAAAGCCGATGTTGCGG | *bla*MIR-1, *bla*ACT-1 |  |
| EBCMR | CTTCCACTGCGGCTGCCAGTT |  |  |
| FOXMF | AACATGGGGTATCAGGGAGATG | *bla*FOX-1 to *bla*FOX-5b |  |
| FOXMR | CAAAGCGCGTAACCGGATTGG |  |  |
| GyrAFQ322 | GAGCTCCTATCTGGATTAT | gyrA | (Komp et al., 2003) |
| GyrAF21 | GAACTCACCCTTCCAGATCCA |  |  |
| ParCF43 | AGCGCCTTGCGTACATGAAT | parC |  |
| ParCR981 | GTGGTAGCGAAGAGGTGGTT |  |  |
| QnrAm-F | AGAGGATTTCTCACGCCAGG | *QnrA* | (Kraychete et al., 2016) |
| QnrAm-R | TGCCAGGCACAGATCTTGAC |  |  |
| QnrSm-F | GCAAGTTCATTGAACAGGGT | *QnrS* |  |
| QnrSm-R | TCTAAACCGTCGAGTTCGGCG |  |  |
| QnrBm-F | GGMATHGAAATTCGCCACTGa | *QnrB* |  |
| QnrBm-R | TTTGCYGYYCGCCAGTCGAAa |  |  |
| PreA | CACCTCATGTTTGAATTCGCC | For *bla*NDM type | This study |
| PreB | CTCTGTCACATCGAAATCGC |  |  |

**Supplementary references**

**References:**

Casella, T., Haenni, M., Madela, N.K., Andrade, L.K.D., Pradela, L.K., and Andrade, L.N.D., et al. (2018). Extended-spectrum cephalosporin-resistant *Escherichia coli* isolated from chickens and chicken meat in Brazil is associated with rare and complex resistance plasmids and pandemic ST lineages. *Journal of Antimicrobial Chemotherapy*. doi: 10.1093/jac/dky335.

Chavda, K.D., Satlin, M.J., Chen, L., Manca, C., Jenkins, S.G., and Walsh, T.J., et al. (2016). Evaluation of a Multiplex PCR Assay To Rapidly Detect Enterobacteriaceae with a Broad Range of β-Lactamases Directly from Perianal Swabs. *Antimicrobial Agents and Chemotherapy.* 60(11), 6957-6961. doi: 10.1128/AAC.01458-16.

Komp, L.P., and Karlsson, A.D. (2003). Mutation rate and evolution of fluoroquinolone resistance in Escherichia coli isolates from patients with urinary tract infections. *Antimicrobial Agents & Chemotherapy* 47(10), 3222. doi: 10.1128/AAC.47.10.3222-3232.2003.

Kraychete, G.B., Botelho, L.A.B., Campana, E.H., Picão, R.C., and Bonelli, R.R. (2016). Updated multiplex PCR for detection of all six plasmid-mediated qnr gene families. *Antimicrobial Agents and Chemotherapy*, 1416-1447. doi: 10.1128/AAC.01447-16.

Perez Perez, F.J., and Hanson, N.D. (2002). Detection of plasmid-mediated AmpC beta-lactamase genes in clinical isolates by using multiplex PCR. *Journal of Clinical Microbiology* 40(6), 2153. doi: 10.1128/JCM.40.6.2153-2162.2002.

Poirel, L., Walsh, T.R., Cuvillier, V., and Nordmann, P. (2011). Multiplex PCR for detection of acquired carbapenemase genes. *Diagnostic Microbiology and Infectious Disease* 70(1), 119-123. doi: 10.1016/j.diagmicrobio.2010.12.002.

Rebelo, A.R., Bortolaia, V., Kjeldgaard, J.S., Pedersen, S.K., Leekitcharoenphon, P., and Hansen, I.M., et al. (2018). Multiplex PCR for detection of plasmid-mediated colistin resistance determinants, *mcr-1, mcr-2, mcr-3, mcr-4* and *mcr-5* for surveillance purposes. *Euro Surveill* 23(6). doi: 10.2807/1560-7917.ES.2018.23.6.17-00672.

**Table S2.** The susceptibility results of 127 *E. coli* isolates for 17 antibiotics.

| Years | Isolates | AM*^a^*P | CZO | CTX | CRO | MEM | AMC | ATM | CT | GEN | AK | CHL | DO | TGC | CIP | ENR | ORB | MAR |
| --- | --- | --- | --- | --- | --- | --- | --- | --- | --- | --- | --- | --- | --- | --- | --- | --- | --- | --- |
| 2012 | UTI-6 | R*^b^* | R | R | R | S*^b^* | S | S | S | R | S | S | R | S | R | R | R | R |
| 2012 | UTI-8 | R | R | R | R | S | R | R | S | R | R | S | R | S | R | R | R | R |
| 2012 | UTI-25 | R | R | R | R | S | R | S | R | R | R | R | R | S | R | R | R | R |
| 2012 | UTI-27 | R | R | R | R | S | S | S | S | S | S | S | S | S | R | R | R | R |
| 2012 | UTI-33 | R | R | S | S | S | R | S | S | S | S | S | R | S | S | S | S | S |
| 2012 | UTI-36 | S | S | S | S | S | S | S | S | S | S | S | S | S | S | S | S | S |
| 2012 | UTI-43 | R | S | S | S | S | S | S | S | S | S | R | R | S | R | R | R | R |
| 2012 | UTI-45 | R | S | S | S | S | S | S | S | S | S | S | S | S | R | R | R | R |
| 2012 | UTI-46 | S | S | S | S | S | S | S | S | S | S | S | S | S | R | R | R | R |
| 2012 | UTI-48 | R | S | S | S | S | S | S | S | S | S | R | R | S | R | R | R | R |
| 2012 | UTI-50 | R | R | R | R | S | R | S | S | S | S | S | S | S | R | R | R | R |
| 2012 | UTI-58 | S | S | S | S | S | S | S | S | R | R | S | S | S | R | R | R | R |
| 2012 | UTI-67 | R | R | S | S | S | R | S | S | S | S | S | S | S | R | R | R | R |
| 2012 | UTI-78 | R | R | R | R | S | S | R | S | S | S | S | S | S | R | R | R | R |
| 2012 | UTI-95 | R | R | R | R | S | S | R | S | R | S | R | R | S | R | S | S | S |
| 2012 | UTI-104 | R | R | S | S | S | S | S | S | S | S | S | S | S | R | R | R | R |
| 2012 | UTI-105 | S | S | S | S | S | S | S | S | S | S | S | R | S | S | S | S | S |
| 2012 | UTI-117 | R | S | S | S | S | S | S | S | S | S | S | R | S | R | S | R | S |
| 2012 | UTI-118 | S | S | S | S | S | S | S | S | S | S | S | S | S | S | S | S | S |
| 2012 | UTI-120 | S | S | S | S | S | S | S | S | S | S | S | S | S | S | S | S | S |
| 2012 | UTI-128 | R | R | R | R | S | S | S | S | R | R | R | R | S | R | R | R | R |
| 2012 | UTI-136 | S | S | S | S | S | S | S | S | S | S | S | S | S | S | S | S | S |
| 2012 | UTI-138 | S | S | S | S | S | S | S | S | S | S | S | S | S | S | S | S | S |
| 2012 | UTR-H3 | R | R | R | R | S | R | R | S | R | S | R | R | S | R | R | R | R |
| 2012 | UTI-R2 | R | R | S | S | S | R | S | R | S | S | S | S | S | S | S | S | S |
| 2012 | UTI-GR | R | R | R | R | S | R | S | S | R | S | S | S | S | R | R | R | R |
| 2012 | UTI-YSY | R | R | R | R | S | S | S | S | S | S | R | R | S | R | S | R | S |
| 2012 | 12CT01 | R | S | S | S | S | S | S | S | R | S | S | R | S | S | S | S | S |
| 2012 | 12DU07 | R | S | S | S | S | S | S | S | R | S | R | R | S | R | R | R | R |
| 2012 | 12DD09 | R | S | S | S | S | S | S | S | S | S | S | R | S | S | S | S | S |
| 2013 | UTI-140 | S | S | S | S | S | S | S | S | S | S | S | S | S | S | S | S | S |
| 2013 | UTI-145 | S | S | S | S | S | S | S | S | S | S | S | S | S | S | S | S | S |
| 2013 | UTI-165 | R | R | R | R | S | S | S | S | R | S | R | R | S | R | R | R | R |
| 2013 | UTI-166 | R | R | R | R | S | R | R | S | R | R | S | S | S | R | R | R | R |
| 2013 | UTI-169 | R | R | R | R | S | S | S | S | R | S | R | R | S | S | S | S | S |
| 2013 | UTI-195 | S | S | S | S | S | S | S | S | S | S | S | R | S | S | S | S | S |
| 2013 | UTI-196 | S | S | S | S | S | S | S | S | S | S | S | R | S | S | S | S | S |
| 2013 | 13DUT01 | S | S | S | S | S | S | S | S | S | S | S | S | S | S | S | S | S |
| 2013 | 13DD01 | R | R | R | R | S | R | R | S | S | S | R | R | S | S | S | S | S |
| 2013 | 13DU12 | R | R | R | R | S | S | R | S | R | S | R | S | S | R | R | R | R |
| 2013 | 15UTI-207 | S | S | S | S | S | S | S | S | R | S | S | S | S | S | S | S | S |
| 2013 | 13DU30 | R | R | R | R | S | S | R | S | R | S | S | R | S | R | R | R | R |
| 2013 | 13DU31 | R | R | R | R | S | R | S | S | S | S | S | R | S | R | S | S | S |
| 2013 | 13DD44 | R | S | S | S | S | S | S | S | S | S | R | R | S | S | S | R | S |
| 2013 | 13DD56 | R | S | S | S | S | R | S | S | S | S | R | R | S | S | S | S | S |
| 2013 | 13DD72 | R | R | R | R | S | S | R | S | S | S | S | S | S | S | S | S | S |
| 2013 | 13DU107 | R | R | R | R | S | R | R | S | R | S | R | R | S | R | R | R | R |
| 2013 | 13DUT132 | R | R | R | R | S | R | R | S | R | S | S | R | S | R | R | R | R |
| 2013 | 13DU135 | R | R | R | R | S | S | R | S | S | S | R | R | S | R | R | R | R |
| 2014 | A41 | R | R | R | R | S | R | R | S | R | R | S | R | S | R | R | R | R |
| 2014 | A42 | R | R | R | R | S | R | R | S | R | S | S | R | S | R | R | R | R |
| 2014 | A46 | R | R | R | R | S | S | R | S | R | R | S | R | S | S | S | S | S |
| 2014 | C38 | R | R | R | R | S | R | R | S | S | S | R | R | S | R | R | R | R |
| 2014 | 14DU18 | S | S | S | S | S | S | S | S | S | S | S | S | S | S | S | S | S |
| 2014 | 14CD33 | R | R | R | R | S | S | R | S | R | S | S | R | S | R | R | R | R |
| 2014 | 14CU80 | R | R | R | R | S | S | R | S | R | S | S | S | S | R | R | R | R |
| 2014 | 14CU87 | R | R | R | R | S | S | R | S | R | R | R | R | S | R | R | R | R |
| 2014 | 14DD94 | S | S | S | S | S | S | S | S | S | S | S | R | S | S | S | S | S |
| 2014 | 14xx104 | R | R | R | R | S | S | R | S | R | S | S | S | S | R | R | R | R |
| 2014 | 14CUT88 | S | S | S | S | S | S | S | S | R | S | S | S | S | S | S | S | S |
| 2014 | 14DF155 | R | S | S | S | S | S | S | S | R | S | S | S | S | S | S | S | S |
| 2014 | 14DU158 | S | S | S | S | S | S | S | S | S | S | S | S | S | S | S | S | S |
| 2014 | UTI-SY1 | R | R | R | R | S | S | R | S | R | S | S | R | S | R | R | R | R |
| 2015 | 15DU8-1 | R | R | R | R | S | R | R | S | R | R | S | R | S | R | R | R | R |
| 2015 | 15DU20 | SS | S | S | S | S | S | S | S | S | S | S | S | S | S | S | S | S |
| 2015 | 15CU32 | R | S | S | S | S | S | S | S | S | S | S | S | S | S | S | S | S |
| 2015 | 15xx149 | R | S | S | S | S | R | S | S | R | S | R | R | S | S | S | R | S |
| 2015 | 15xx158 | R | R | R | R | S | R | R | S | R | R | S | R | S | R | R | R | R |
| 2015 | 15CU184 | R | R | R | R | S | S | R | S | R | R | R | R | S | R | R | R | R |
| 2015 | 15CF186 | R | R | R | R | S | S | R | S | R | R | R | R | S | R | R | R | R |
| 2015 | 15DU193 | R | R | R | R | S | R | R | S | R | R | R | R | S | R | R | R | R |
| 2015 | 15DU197 | R | R | R | R | S | R | R | S | R | S | S | S | S | S | S | R | S |
| 2015 | 15DD198 | R | S | S | S | S | S | S | S | R | S | S | R | S | S | S | S | S |
| 2015 | UTI-50 | R | R | R | R | S | S | R | S | S | R | S | S | S | S | S | S | S |
| 2015 | UTI-209 | R | R | R | R | S | S | S | S | R | S | S | R | S | R | R | R | R |
| 2015 | UTI-213 | R | R | R | R | S | S | R | S | S | S | S | S | S | R | R | R | R |
| 2015 | UTI-219 | R | R | R | R | S | S | R | S | S | S | S | S | S | S | R | R | R |
| 2016 | 16DU02 | R | R | R | R | R | R | R | S | R | R | R | S | S | R | R | R | R |
| 2016 | 16DF03 | R | R | R | R | R | R | R | S | R | R | R | S | S | R | R | R | R |
| 2016 | A9 | R | R | R | R | S | R | R | S | R | R | R | R | S | R | R | R | R |
| 2016 | A28 | R | R | R | R | S | R | R | S | R | S | R | R | S | R | R | R | R |
| 2016 | B1 | R | R | R | R | S | R | R | S | R | S | S | R | S | R | R | R | R |
| 2016 | B7 | R | R | R | R | S | R | R | S | R | R | R | R | S | R | R | R | R |
| 2016 | C36 | R | R | R | R | S | R | R | S | R | R | S | R | S | R | R | R | R |
| 2016 | C37 | R | R | S | S | S | S | S | S | S | S | R | R | S | R | R | R | R |
| 2016 | C40 | R | R | R | R | S | S | R | S | R | S | R | S | S | S | S | S | S |
| 2016 | D29 | R | R | S | S | S | R | S | S | R | S | R | S | S | R | R | R | S |
| 2016 | R7 | R | R | R | R | S | S | R | S | R | R | R | R | S | R | R | R | R |
| 2016 | R8 | R | R | R | R | R | R | R | S | R | R | S | R | S | R | R | R | R |
| 2016 | L224 | S | S | S | S | S | S | S | S | S | S | S | S | S | S | S | S | S |
| 2016 | 16xx04 | S | S | S | S | S | S | S | S | S | S | S | S | S | S | S | S | S |
| 2016 | VTH08 | R | R | R | R | S | R | R | S | S | S | S | S | S | R | S | R | S |
| 2016 | VTH021 | R | R | R | R | S | S | R | R | R | S | R | R | S | R | R | R | R |
| 2016 | VTH026 | S | S | S | S | S | S | S | S | S | S | S | S | S | S | S | S | S |
| 2016 | VTH032 | S | S | S | S | S | S | S | S | S | S | S | S | S | S | S | S | S |
| 2016 | VTH035 | R | R | R | R | S | S | R | S | S | S | R | R | S | R | R | R | R |
| 2016 | VTH039 | R | R | R | R | S | S | S | S | R | S | R | R | S | S | S | S | S |
| 2016 | VTH063 | R | R | R | R | S | R | S | S | S | S | S | S | S | R | S | S | S |
| 2016 | VTH068 | R | R | S | S | S | S | S | S | S | S | S | R | S | S | S | S | S |
| 2017 | VTH071 | S | S | S | S | S | S | S | S | R | S | S | S | S | R | R | R | R |
| 2017 | VTH082 | R | R | R | R | S | S | S | S | R | S | R | R | S | R | R | R | R |
| 2017 | VTH090 | R | R | R | R | S | S | S | R | R | S | R | R | S | R | R | R | R |
| 2017 | VTH091 | R | R | R | R | S | S | S | S | R | R | R | R | S | R | R | R | R |
| 2017 | 17DD3 | R | R | R | R | S | R | S | S | R | R | S | R | S | R | R | R | R |
| 2017 | 17DU12 | R | R | R | R | S | R | S | S | R | S | S | R | S | R | R | R | R |
| 2017 | 17DUT19 | S | S | S | S | S | S | S | S | R | S | S | R | S | S | S | S | S |
| 2017 | 17DU21 | R | R | R | R | S | R | R | S | R | S | S | S | S | S | S | S | S |
| 2017 | 17DF22 | S | S | S | S | S | S | S | S | S | S | S | S | S | S | S | S | S |
| 2017 | 17DU23 | R | R | S | S | S | R | S | S | S | S | S | S | S | S | S | S | S |
| 2017 | 17DU26 | R | R | S | S | S | R | S | S | R | S | R | R | S | R | R | R | R |
| 2017 | 17DUT30 | R | R | R | R | S | R | R | R | S | S | R | R | S | S | R | R | S |
| 2017 | 17DU31 | R | R | S | S | S | S | S | S | S | S | S | S | S | R | R | R | R |
| 2017 | 17DU33 | R | R | S | S | S | R | S | S | S | S | S | S | S | S | S | S | S |
| 2017 | 17DU34 | R | R | R | R | S | R | R | S | S | S | S | R | S | R | R | R | R |
| 2017 | 17DU35 | R | R | R | R | S | R | R | S | R | S | R | R | S | R | R | R | R |
| 2017 | 17DU36 | R | R | S | S | S | S | S | S | S | S | S | S | S | S | S | S | S |
| 2017 | 17CUT37 | S | S | S | S | S | S | S | S | S | S | S | S | S | S | S | S | S |
| 2017 | 17DU38 | R | R | R | R | S | S | S | S | S | S | S | S | S | S | S | S | S |
| 2017 | 17DU39 | R | R | R | R | S | R | R | S | R | S | S | R | S | R | R | R | R |
| 2017 | 17DU40 | R | R | R | R | S | R | R | S | R | S | S | R | S | R | R | R | R |
| 2017 | 17DU41 | R | R | R | R | S | S | R | S | R | S | S | R | S | R | R | R | R |
| 2017 | 17DU42 | R | R | R | R | R | R | R | S | R | R | S | R | S | R | R | R | R |
| 2017 | VTH125 | S | S | S | S | S | S | S | S | S | S | S | S | S | S | S | S | S |
| 2017 | VTH136 | R | R | R | R | S | R | R | S | R | S | R | R | S | R | R | R | R |
| 2017 | VTH142 | R | R | R | R | S | R | R | S | R | R | R | R | S | R | R | R | R |
| 2017 | VTH147 | R | R | R | R | S | R | R | S | R | R | R | R | S | R | R | R | R |
| 2017 | VTH152 | S | S | S | S | S | S | S | S | S | S | S | S | S | S | S | S | S |

*^a^* Abbreviation annotation: AMP, Ampicillin; CZO, Cefazolin; CTX, Cefotaxime; CRO, Ceftriaxone; MEM, Meropenem ; AMC, Amoxicillin + Clavulanate; ATM, Aztreonam; CT, Colistin; GEN, Gentamycin; AK, Amikacin; CHL, Chloramphenicol; DO, Doxycycline; TGC, Tigecycline; CIP, Ciprofloxacin; ENR, Enrofloxacin; ORB, Orbifloxacin; MAR, Marbofloxacin.

*^b^* “R” represent resistance, “S” represent susceptibility. Here, Intermediary was defined as resistance.

Table S3 Resistance rates (%) of 17 antibiotics, no classified, from 2012-2017.

| Antibiotics | Resistance rates (%) *^a^* | | | | | | |
| --- | --- | --- | --- | --- | --- | --- | --- |
|  | 2012 | 2013 | 2014 | 2015 | 2016 | 2017 | Total *^b^* |
| Ampicillin | 73.33 | 68.42 | 71.43 | 92.86 | 86.36 | 78.57 | 77.95 |
| Cefazolin | 43.33 | 57.89 | 64.29 | 71.43 | 86.36 | 75.00 | 65.35 |
| Cefotaxime | 36.67 | 57.89 | 64.29 | 71.43 | 72.73 | 60.71 | 58.27 |
| Ceftriaxone | 36.67 | 57.89 | 64.29 | 71.43 | 72.73 | 60.71 | 58.27 |
| Amoxicillin+  Clavulanate | 10.00 | 26.32 | 14.29 | 14.29 | 27.27 | 39.29 | 22.83 |
| Aztreonam | 13.33 | 31.58 | 71.43 | 57.14 | 59.09 | 39.29 | 40.94 |
| Ciprofloxacin | 60.00 | 36.84 | 57.14 | 50.00 | 59.09 | 60.71 | 55.12 |
| Enrofloxacin | 56.67 | 36.84 | 57.14 | 50.00 | 59.09 | 60.71 | 54.33 |
| Orbifloxacin | 63.33 | 36.84 | 57.14 | 64.29 | 59.09 | 60.71 | 57.48 |
| Marbofloxacin | 53.33 | 36.84 | 57.14 | 50.00 | 59.09 | 57.14 | 52.76 |
| Gentamycin | 33.33 | 42.11 | 71.43 | 64.29 | 59.09 | 60.71 | 52.76 |
| Amikacin | 13.33 | 5.26 | 21.43 | 42.86 | 36.36 | 17.86 | 21.26 |
| Chloramphenicol | 26.67 | 42.11 | 14.29 | 35.71 | 59.09 | 32.14 | 35.43 |
| Doxycycline | 30.00 | 52.63 | 50.00 | 28.57 | 50.00 | 46.43 | 42.52 |
| Tigecycline | 0.00 | 0.00 | 0.00 | 0.00 | 0.00 | 0.00 | 0.00 |
| Colistin | 6.67 | 0.00 | 0.00 | 0.00 | 4.55 | 7.14 | 3.94 |
| Meropenem | 0.00 | 0.00 | 0.00 | 0.00 | 13.64 | 0.00 | 2.36 |

*^a^*The numbers of isolates were 30, 19, 14, 14, 22 and 28 from 2012~2017, respectively. Resistance break point for every antibiotic was adopted and resistance results were interpreted based on CLSI.

*^b^*“Total” represent the overall resistance rates of 127 isolates.

Table S4. Multidrug resistance rates of 127 isolates based on antimicrobial category classified from 2012~2017.

| Year | Multidrug resistance rates (%) | | | |
| --- | --- | --- | --- | --- |
|  | 0~2 antibiotics*^a^* | 3~5 antibiotics | 6~9 antibiotics | ≥ 3 antibiotics |
| 2012（n=30） | 33.33 | 40.00 | 26.67 | 66.67 |
| 2013（n=19） | 31.58 | 15.79 | 52.63 | 68.42 |
| 2014（n=14） | 35.71 | 0.00 | 64.29 | 64.29 |
| 2015（n=14） | 14.29 | 28.57 | 57.14 | 85.71 |
| 2016（n=22） | 18.18 | 13.64 | 68.18 | 81.82 |
| 2017（n=28） | 25.00 | 14.29 | 60.71 | 75.00 |

*^a^*Antibiotics were classified as: Penicillins (Ampicillin), Penicillins + β-lactamase inhibitors (Amoxicilline + Clavulanate), Non-extended spectrum cephalosporins (Cefazoline), Extended-spectrum cephalosporins (Cefotaxime and Ceftriaxone), Carbapenems (Meropenem), Monobactams (Aztreonam), Polymyxins (Colistin), Aminoglycosides (Gentamycin and Amikacin), Phenicols (Chloramphenicol), Tetracyclines (Doxycyline), Glycylcyclines (Tigecycline), and quinolones (Ciproxacin, Enrofloxacin, Orbifloxacin, and Marbofloxacin)

Table S5. Multidrug resistance rates of 127 isolates from 2012-2017.

| Year | Resistance rates (%) | | |
| --- | --- | --- | --- |
|  | 0~2 antibiotics *^b^* | 3~10 antibiotics | 11~17 antibiotics |
| 2012（n=30） | 40.0%（12/30） | 46.6%（14/30） | 13.3%（4/30） |
| 2013（n=19） | 31.6%（6/19） | 36.8%（7/19） | 31.6%（6/19） |
| 2014（n=14） | 35.7%（5/14） | 28.6%（4/14） | 35.7%（5/14） |
| 2015（n=14） | 21.4%（3/14） | 42.9%（6/14） | 35.7%（5/14） |
| 2016（n=22） | 22.7%（5/22） | 27.3%（6/22） | 50.0%（11/22） |
| 2017（n=28） | 28.6%（8/28） | 17.9%（5/28） | 53.6%（15/28） |

*^a^* The numbers of isolates were 30, 19, 14, 14, 22 and 28 from 2012~2017, respectively.

*^b^* Three resistance group were classified based on amounts of antibiotic rather than antibiotic class.

Table S6. Mutation of gyrA and parC in 77 quinolone-resistant isolates.

| genes | gyrA | | | parC | | |
| --- | --- | --- | --- | --- | --- | --- |
| Mutation sites | 83 | 87 | Isolates numbers | 80 | 84 | Isolates numbers |
| Amino acid substitutions | *Ser (S)* | *Asp (D)* | 13 | *Ser (S)* | *Glu (E)* | 33 |
|  | Leu (L) | Asn (N) | 38 | I | *Glu (E)* | 22 |
|  | Leu (L) | Tyr (Y) | 9 | I | Gly (G) | 14 |
|  | *Ser (S)* | Asn (N) | 2 | I | Val (V) | 8 |
|  | *Ser (S)* | Gly (G) | 3 | - | - | - |
|  | Leu (L) | *Asp (D)* | 12 | - | - | - |

**Figure S**

**Figure S1**. Antibiotic resistance trends for the indicated antibiotics in dogs and cats from 2012 to 2017.


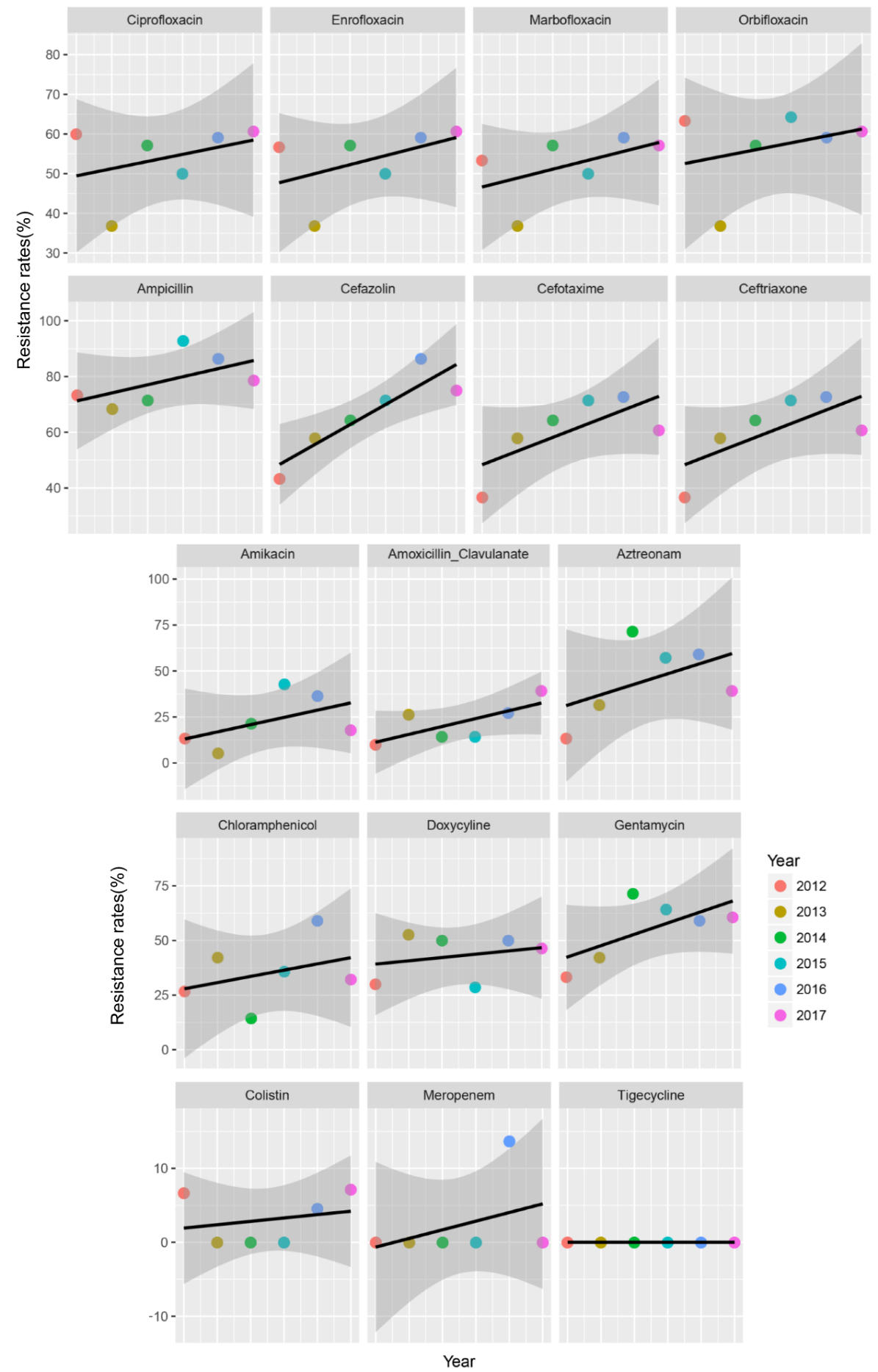


**Figure S2.** MDR prevalence in *E. coli* isolates from dogs and cats in Beijing, China (2012–2017). Group A: isolates resistant to 0–2 antibiotics, Group B: isolates resistant to 3–10 antibiotics; Group C: isolates resistant to 11–17 antibiotics. Three resistance group were classified based on amounts of antibiotic rather than antibiotic class.


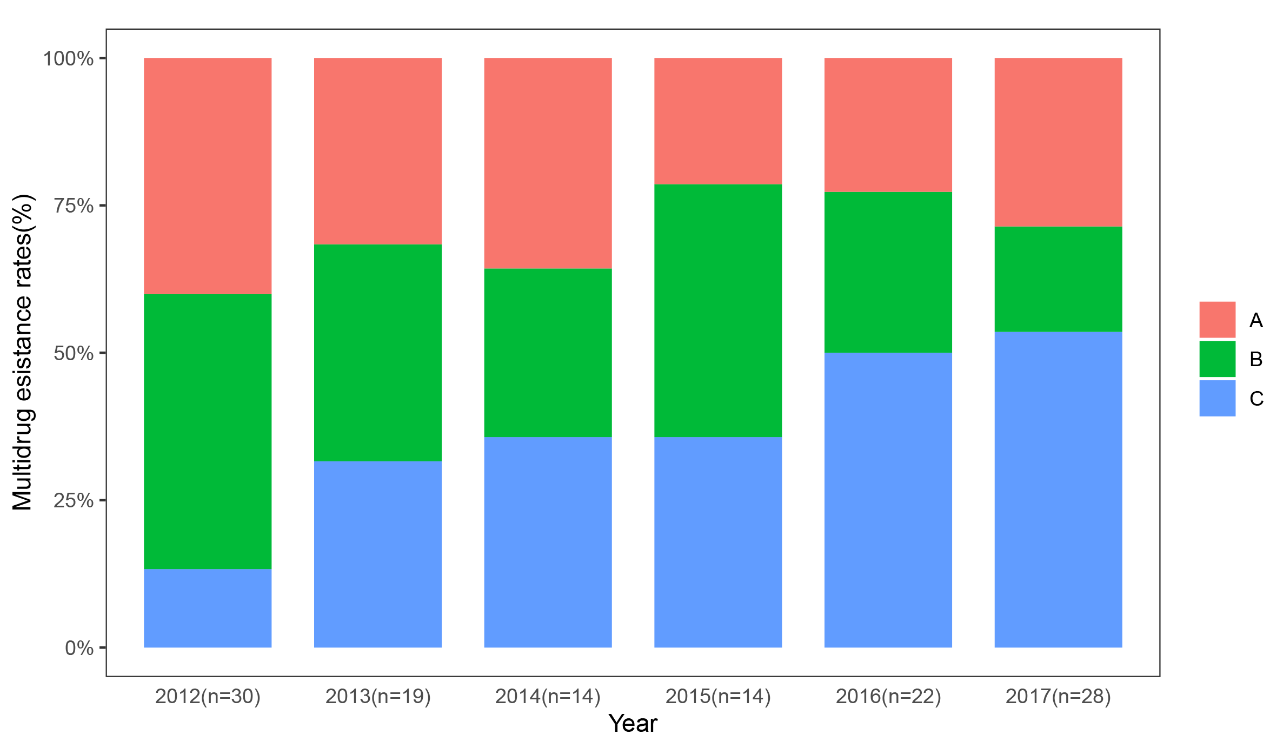


**Figure S3.** Antibiotic dose and resistance rates for clinical *E coli* isolates from the Veterinary Teaching Hospital of China Agricultural University (January 2014 to Sep 2017).
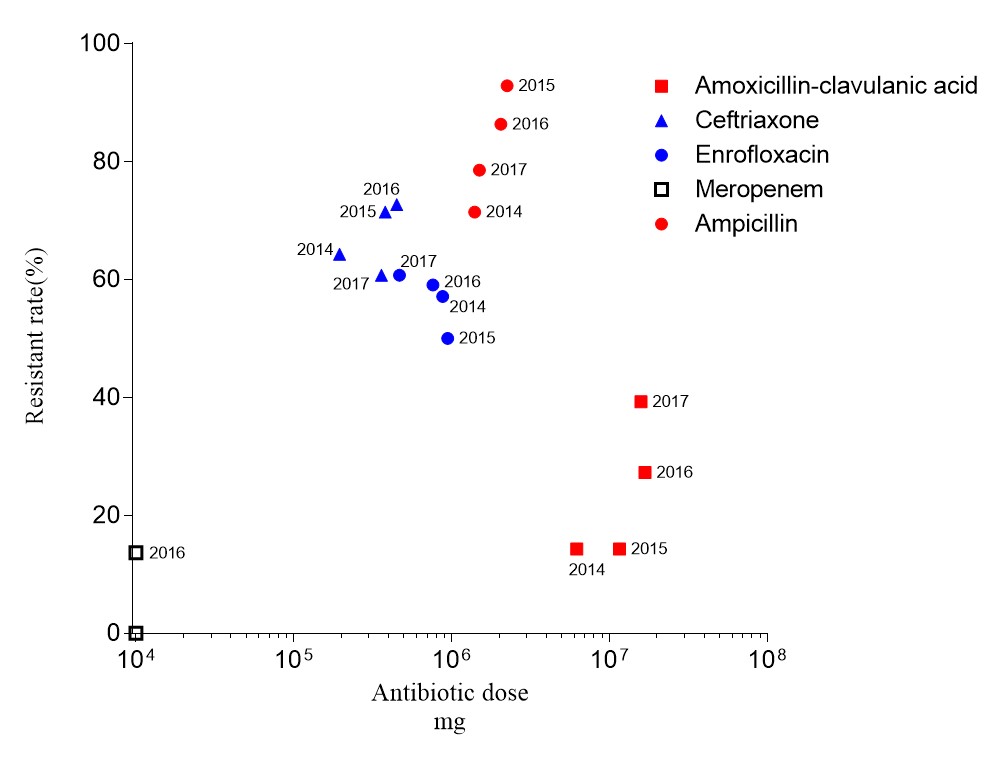


**Figure S4**. Resistance genes isolate rates. A total of 10 genes type among 127 strains were analyzed.


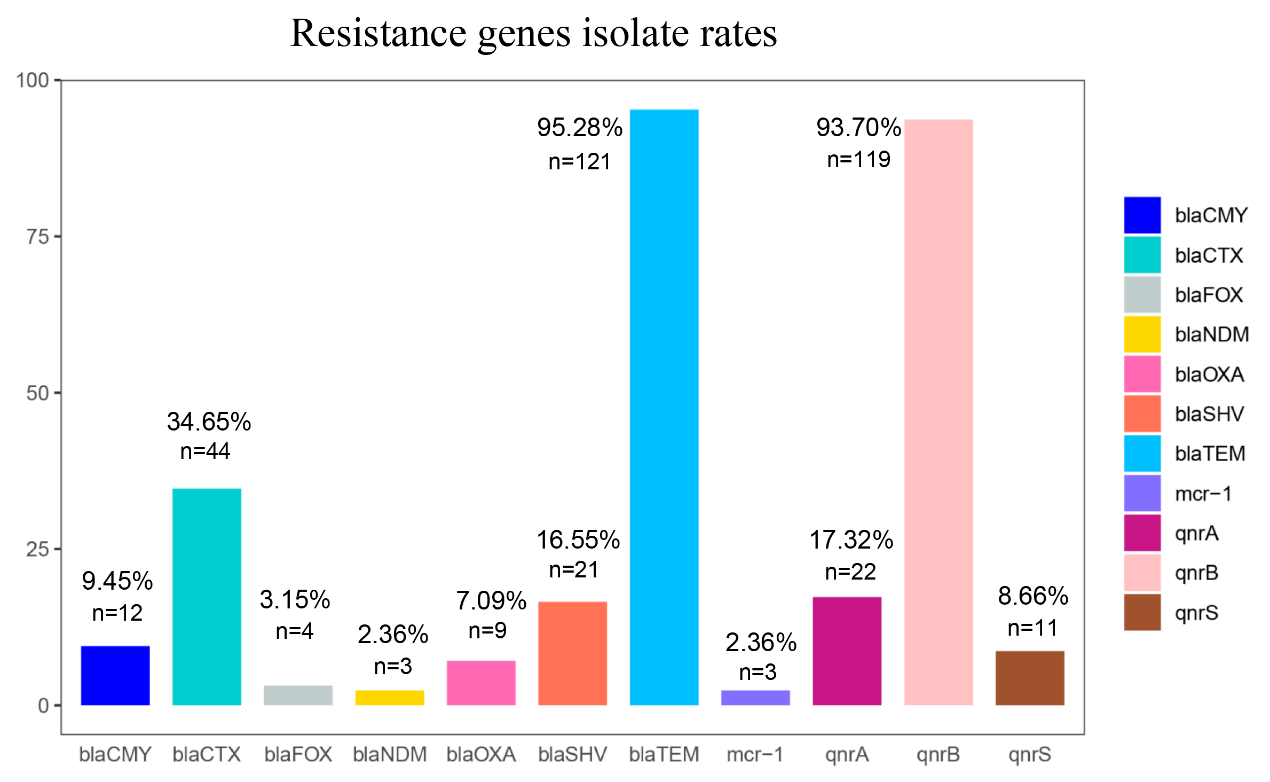


**Figure S5.** The heatmap of MDR. a and b were MDR of *bla*_CTX-M_-positive (n=44) and negative(n=73) *E. coli*, respectively. The cyan and hotpink represent resistance and susceptibility, respectively.

a


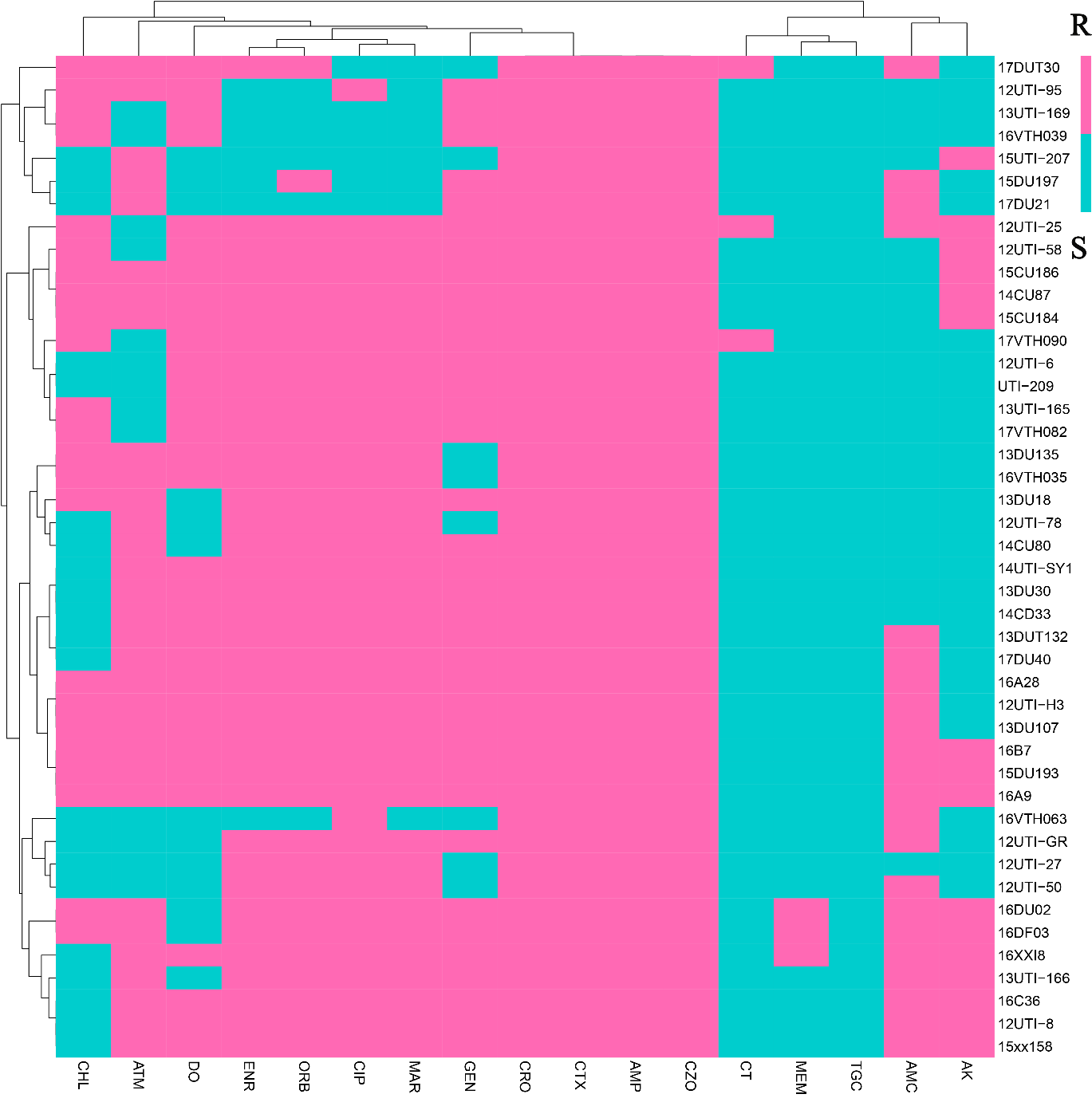


b


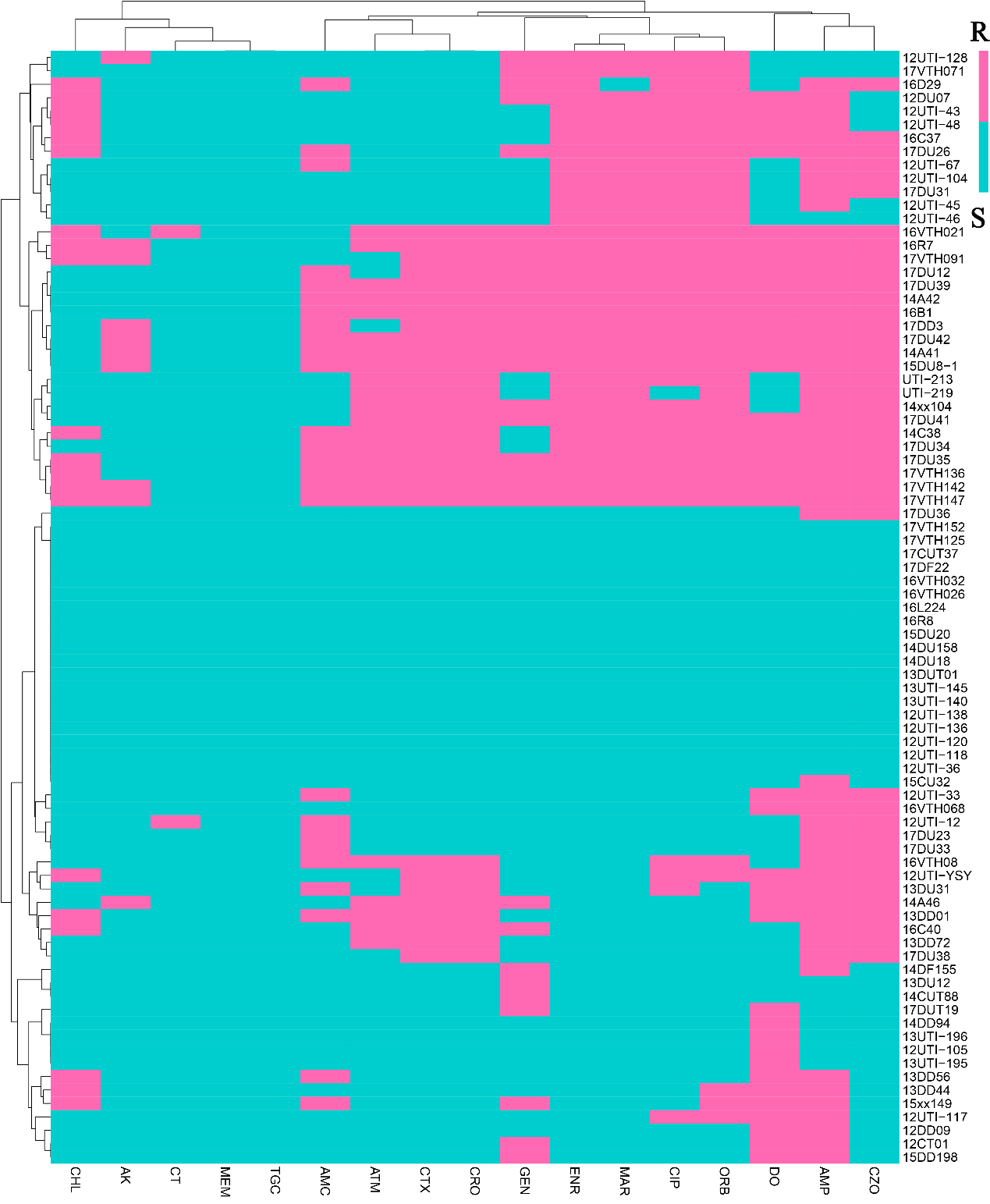


**Figure S6**. The comparison of NDM-5 carrying plasmid sequences. Each color represents an NDM-5 carrying plasmid. The internal ring is the reference sequence of NDM-1 carrying plasmid pNDM_MGR194, and the outside rings are other 9 plasmids, including pP16NDM-502 and pP16NDM-503 from this study. From inside to outside, the rings were p1079-NDM (MG825384), pAD-19R, pCRCB-101_1, pL65-9, pNDM_MGR194, pQDE2-NDM, pZHDC40, pP16NDM-502 and pP16NDM-503.


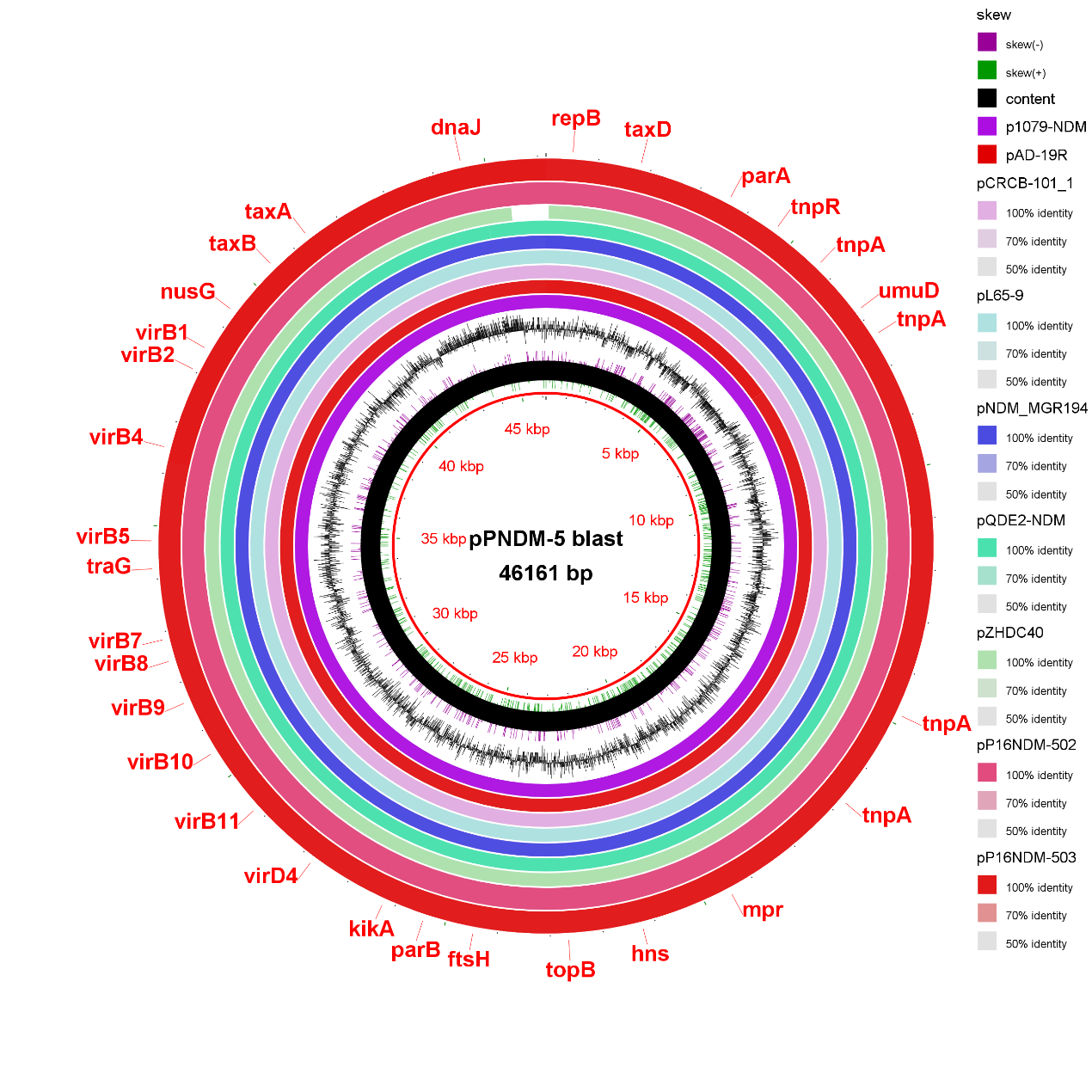

Supplement: Supplementary file 1 [file Table_1.docx]
